# Evaluation of Kenya’s readiness to transition from sentinel surveillance to routine HIV testing for antenatal clinic-based HIV surveillance

**DOI:** 10.1186/s12879-016-1434-1

**Published:** 2016-03-05

**Authors:** Martin Sirengo, George W. Rutherford, Boaz Otieno-Nyunya, Timothy A. Kellogg, Davies Kimanga, Nicholas Muraguri, Mamo Umuro, Joy Mirjahangir, Ellen Stein, Margaret Ndisha, Andrea A. Kim

**Affiliations:** Ministry of Health, National AIDS and STI Control Programme, Kenyatta National Hospital Grounds, 19361-00202 Nairobi, Kenya; Global Health Sciences, University of California, San Francisco, California USA; Division of Global HIV and Tuberculosis, Center for Global Health, Centers for Disease Control and Prevention, Nairobi, Kenya; Ministry of Health, National Public Health Laboratory Services, Nairobi, Kenya; Kenya Medical Research Institute, Nairobi, Kenya

**Keywords:** HIV, Kenya, Perinatal transmission, Pregnant women, Surveillance

## Abstract

**Background:**

Sentinel surveillance for HIV among women attending antenatal clinics using unlinked anonymous testing is a cornerstone of HIV surveillance in sub-Saharan Africa. Increased use of routine antenatal HIV testing allows consideration of using these programmatic data rather than sentinel surveillance data for HIV surveillance.

**Methods:**

To gauge Kenya’s readiness to discontinue sentinel surveillance, we evaluated whether recommended World Health Organization standards were fulfilled by conducting data and administrative reviews of antenatal clinics that offered both routine testing and sentinel surveillance in 2010.

**Results:**

The proportion of tests that were HIV-positive among women aged 15–49 years was 6.2 % (95 % confidence interval [CI] 4.6–7.7 %] in sentinel surveillance and 6.5 % (95 % CI 5.1–8.0 %) in routine testing. The agreement of HIV test results between sentinel surveillance and routine testing was 98.0 %, but 24.1 % of specimens that tested positive in sentinel surveillance were recorded as negative in routine testing. Data completeness was moderate, with HIV test results recorded for 87.8 % of women who received routine testing.

**Conclusions:**

Additional preparation is required before routine antenatal HIV testing data can supplant sentinel surveillance in Kenya. As the quality of program data has markedly improved since 2010 a repeat evaluation of the use of routine antenatal HIV testing data in lieu of ANC sentinel surveillance is recommended.

## Background

Sentinel surveillance (SS) using unlinked anonymous HIV testing (UAT) of women attending antenatal clinics (ANC) provides a primary source of data on HIV prevalence in the adult population in countries with generalized HIV epidemics [1, 2]. ANC SS conducted over many years in the same setting allows for monitoring of trends in HIV prevalence. When restricted to women aged 15–19 or 15–24 years who are assumed to have been recently infected, HIV prevalence from ANC SS can be used as a proxy for estimating trends in HIV incidence [1]. Despite these advantages, ANC SS has several drawbacks, including the inability to give results and therapy to HIV- infected women, non-random selection of participating clinics, overrepresentation of urban clinics and restriction to only women attending ANC. ANC SS excludes women who are not pregnant for reasons of reduced fertility [3] or contraception, who are not sexually active, who do not seek care during pregnancy, or who receive antenatal care at home. Additionally, ANC SS is a discrete operation and not part of routine clinical care. As such, it requires the mobilization of significant human and monetary resources at the national level.

Several countries have begun using routinely collected HIV testing data from ANC to estimate HIV prevalence among pregnant women. The World Health Organization (WHO) recommends that five areas be evaluated before a successful transition from ANC SS to surveillance using routine antenatal HIV testing can be considered [3]. These are (1) agreement between ANC SS and routine antenatal HIV test results, (2) magnitude of selection bias in routine antenatal testing compared to ANC SS data, (3) all ANC SS sites should provide routine antenatal HIV testing, (4) the quality of routinely collected antenatal data and (5) the state of routine HIV testing quality assurance practices [4]. To assess Kenya’s readiness to transition from ANC SS to using routinely collected antenatal HIV testing data to monitor trends in HIV prevalence among pregnant women, we conducted a cross-sectional assessment in all 43 ANC sites participating in the 2010 round of ANC SS. 

## Methods

ANC SS was conducted in a non-random sample of 43 (1 %) of Kenya’s 4,400 antenatal facilities, which were selected to include populations from areas that were rural (*n* = 15), urban (*n* = 13) and a mixture of rural and urban areas (*n* = 15). We compared data from a sample from the ANC registry (routine antenatal HIV testing) and from ANC SS in these clinics. The ANC registry sample consisted of women who attended one of the clinics for their first prenatal visit of their current pregnancy. We sampled up to 400 consecutive women during the 3 months prior to ANC SS (March, April, and May 2010) and 1 month after ANC SS (October 2010) if the sample size was not reached during the initial sampling period. Trained field workers used programmed personal data assistants (PDAs) to collect data from the ANC register or a dedicated PMTCT registry. For routine ANC testing, HIV testing was performed using up to three third-generation rapid HIV tests: 1) Determine (Alere, Inc., Waltham, Massachusetts, USA) as the screening test; 2) if reactive on Determine, SD Bioline (Standard Diagnostics, Inc. Yongin-si, South Korea) was used as the confirmatory test; and 3) if non-reactive on SD Bioline, Uni-Gold Recombigen HIV (Trinity Biotech PLC, Bray, Ireland) was used as the tie-breaker test.

Based on WHO’s recommendations on conducting sentinel HIV serosurveys among pregnant women [2], for the ANC SS sample, we included the first 400 consecutive women seen in urban clinics and the first 300 seen in rural and mixed urban-rural clinics for their first prenatal visit of their current pregnancy during the ANC SS period (June through September 2010). Remaining blood from routine syphilis testing was tested centrally for HIV antibodies using a fourth-generation enzyme linked immunoassay (EIA) (Vironostika® HIV Uni-Form II Ag/Ab, bioMérieux SA, Marcy l’Etoile, France), and reactive results were confirmed using a third-generation EIA (Murex HIV.1.2.O, Murex Biotech, Ltd., Dartford, UK).

We used ANC SS data forms to abstract demographic, obstetrical, clinical, and UAT data for each patient enrolled during the ANC SS period. We analyzed data from the participating ANCs to estimate the proportion of tests that were HIV-positive among women seen for the first visit of the current pregnancy using routine HIV testing data recorded in the ANC registry sample and UAT results from the ANC SS sample. We also conducted administrative reviews at each of the participating clinics. We assessed whether ANC SS sites provided routine antenatal HIV testing, the completeness of various data elements included in routine ANC registers and ANC SS forms, and reviewed the results of the June 2010 and October 2010 rounds of the National Proficiency Testing (PT) Program for participating clinics.

To assess comparability of estimates from ANC SS and routine antenatal testing, we used independent samples to compare the aggregate estimates of the proportion of tests that were HIV-positive obtained from routine ANC registers to those obtained from ANC SS forms using the z-test. When reporting the proportion of tests that were positive from ANC registers, we used weighted estimates that accounted for facility-specific sample size differences. We compared estimates of the proportion of tests that were positive among 15-to-49-year-old women obtained from routine ANC registers and ANC SS using z-tests. We calculated 95 % Wald confidence intervals (CI) around estimates of the proportion of tests that were HIV-positive and recorded interquartile ranges (IQR) around medians. When reporting confidence intervals and p-values, we adjusted standard errors for potential clustering on ANC facility.

To assess the level of agreement between individual-level test results from routine HIV test results and UAT results for the same patient seen during the sentinel surveillance period, we used the UAT results as the reference to calculate the percent agreement between test results. A positive percent agreement ranging from 94.7 %-98.6 % and negative percent agreement ranging from 99.7 %-99.9 % were considered acceptable based on WHO-recommended benchmarks [3].

We calculated additional statistics to assess potential bias in the HIV prevalence estimates from routine HIV testing. The first was percent bias or the percent change (positive or negative) from the total HIV prevalence measured in ANC SS to the observed HIV prevalence in routinely tested women. This was calculated as the difference between the HIV prevalence among women sampled in ANC SS who consented to routine HIV testing and the overall ANC SS prevalence divided by the overall ANC SS prevalence. A percent bias between -10 % and +10 % at all ANC SS sites was considered sufficiently accurate to use for HIV surveillance purposes [3]. Two additional statistics were conducted to explore non-consent bias. The first was the uptake rate of routine HIV testing, calculated as the proportion of pregnant women sampled in ANC SS that received routine HIV testing. The second was the differential prevalence ratio defined as the ratio of the HIV prevalence in women sampled in ANC SS who received routine HIV testing to the prevalence for women sampled in ANC SS who did not receive routine HIV testing. The closer this ratio was to 1.0, the more similar the prevalence between women who received and did not receive routine HIV testing [3].

The study was reviewed and approved as research that does not include identifiable human subjects by the US Centers for Disease Control and Prevention (CDC) and did not require Institutional Review Board review for human experimentation. The study was also approved by the Ethical Review Committee of the Kenya Medical Research Institute and the Committee on Human Research of the University of California, San Francisco. The data supporting the conclusions of this article are included within the article. The full study dataset is available upon request.

## Results

We abstracted a total of 14,795 ANC registry records for our study and 13,926 ANC SS forms. The majority of women were aged 20–29 years and married. The clinics were evenly distributed between rural, urban and mixed urban/rural areas.

1. Assessment of WHO standard 1: Agreement between ANC sentinel surveillance and routine antenatal HIV test results

The proportion of women recorded in the ANC registry sample who had a HIV-positive result from routine antenatal HIV testing was 6.5 % (95 % CI, 5.1–8.0 %) and among those surveyed in the ANC SS sample was 6.2 % (95 % CI, 4.6–7.7 %, *p* = 0.21). The median HIV proportion positive in the 43 clinics were similar (5.4 % in routine HIV testing, IQR 3.0–7.5 %, and 4.6 % in ANC SS, IQR 3.2–7.6 %, *p* = 0.44). In nearly all demographic categories the prevalence estimates were similar and had overlapping confidence intervals (Table 1).

**Table 1:** HIV prevalence estimates from ANC registers and ANC sentinel surveillance sites by demographic characteristics, Kenya, 2010

Variable	Antenatal clinic register			Antenatal clinic sentinel surveillance sites			Prevalence Ratio PMTCT/ANC SS
	Unweighted		Weighted HIV prevalence_*a*_ (95 % CI_*b*_)	HIV+	Total	HIV prevalence_*a*_ (95 % CI_*b*_)	
	HIV+	Total					
Overall	884	13064	6.5 (5.1, 8.0)	846	13,745	6.2 (4.6, 7.7)	1.1
Age group (years)							
15–19	59	1898	3.1 (2.2, 3.9)	62	1754	3.5 (2.2, 4.9)	0.9
20–24	279	4941	5.5 (4.0, 7.0)	253	5051	5.0 (3.6, 6.4)	1.1
25–29	260	3377	7.3 (5.1, 9.6)	249	3694	6.7 (4.8, 8.7)	1.1
30–34	193	1861	9.9 (7.6, 12.2)	190	2083	9.1 (6.7, 11.5)	1.1
35–39	73	806	8.9 (6.5, 11.3)	70	892	7.9 (5.3, 10.4)	1.1
40–49	20	181	10.4 (6.0, 14.9)	22	271	8.1 (4.2, 12.1)	1.3
15–24	338	6501	4.8 (3.6, 6.1)	315	6805	4.6 (3.3, 5.9)	1.0
Marital status							
Single	79	1251	6.3 (4.6, 8.1)	117	1669	7.0 (5.6, 8.4)	0.9
Married	788	11637	6.5 (5.0, 8.0)	709	11,904	6.0 (4.4, 7.5)	1.1
Separated/divorced	7	64	9.3 (2.1, 16.4)	5	72	6.9 (0.98, 12.9)	1.3
Widowed	6	25	26.7 (9.0, 44.3)	11	30	36.7 (16.3, 57.0)	0.7
Missing	4	87	5.7 (0.0, 13.1)	4	70	5.7 (0.15, 11.3)	1.0
Gravidity							
One	146	3968	3.6 (2.7, 4.6)	149	4344	3.4 (2.6, 4.3)	1.1
Two	259	3562	7.0 (5.4, 8.7)	236	3710	6.4 (4.6, 8.1)	1.1
Three	219	2364	9.0 (7.0, 11.0)	211	2479	8.5 (6.2, 10.8)	1.1
Four or more	258	3138	8.0 (5.3, 10.7)	248	3163	7.8 (5.2, 10.4)	1.0
Facility setting							
Rural	319	3863	7.6 (3.46, 11.7)	246	3845	6.4 (2.8, 10.0)	1.2
Urban	367	4618	8.0 (6.2, 9.7)	406	4971	8.2 (5.8, 10.6)	1.0
Mixed	198	4583	4.3 (3.2, 5.4)	194	4929	3.9 (2.9, 4.9)	1.1
Region							
Central	49	1125	3.7 (1.7, 5.6)	49	1419	3.5 (2.5, 4.4)	1.1
Coast	64	1554	4.2 (1.8, 6.7)	65	1510	4.3 (2.6, 6.0)	1.0
Eastern	64	1268	5.0 (4.1, 5.9)	61	1507	4.1 (2.6, 5.5)	1.2
North Eastern	3	368	0.82 (0.0, 1.7)	8	392	2.0 (0.89, 4.0)	0.4
Nairobi	150	1975	7.7 (5.9, 9.4)	184	2232	8.2 (5.1, 11.4)	0.9
Nyanza	255	1537	15.6 (8.7, 22.6)	233	1509	15.4 (9.3, 21.9)	1.0
Rift Valley	154	3427	4.5 (3.5, 5.4)	159	3449	4.6 (3.5, 5.7)	1.0
Western	145	1810	8.1 (5.2, 11.1)	87	1727	5.0 (3.0, 7.1)	1.6

Abbreviations: *ANC* antenatal clinic, *PMTCT* prevention of mother-to-child transmission, *CI* 95 % confidence intervals

_*a*_Prevalence estimates are percentages and exclude records that were missing HIV test results

_*b*_We calculated 95 % CI for PMTCT using the Wald asymptotic CI and for ANC sentinel surveillance using the exact CI

Of 12,873 negative UAT results, 84 (0.7 %) were recorded as positive on routine HIV testing. Of 818 HIV-positive UAT results, 197 (24.1 %) were recorded as negative based on routine HIV testing. The overall positive percent agreement between the routine HIV test result and UAT result was 75.9 %. In contrast, the overall negative percent agreement was 99.4 %.

2. Assessment of WHO standard 2: Magnitude of non-consent bias in routine antenatal testing

Overall 5.9 % of women who consented to routine antenatal HIV testing and were also tested in ANC SS had HIV-positive tests. Conversely, 6.2 % of all women tested in ANC SS tested HIV-positive, corresponding to a percent bias of -3.4 % (data not shown). By site, the bias ranged from -58.9 % to +6.3 %, with six clinics falling below <-10 %. Overall the consent rate, including women with known HIV infection who were not retested, was 98.7 %, and the differential prevalence ratio was 3.7. At the site level, the median consent rate was 99.5 %, ranging from a low of 92.4 % to a high of 100.0 %. Overall the consent rate, including women with known HIV infection who were not retested, was 98.7 %, and the differential prevalence ratio was 3.7.

3. Assessment of WHO standard 3: All ANC sentinel sites should provide routine antenatal HIV testing

Routine antenatal HIV testing was available at all 43 sentinel surveillance sites. However several clinics had participated in ANC SS for many years prior to the implementation of routine antenatal HIV testing.

4. Assessment of WHO standard 4: Quality of routinely collected antenatal data

The completeness of data was higher in ANC SS than in ANC registers. That is, forms completed during ANC SS had a smaller proportion of missing values than did the routine ANC registers. Syphilis test results were nearly 100 % complete in ANC SS but present in just over half of the records in the ANC registers. Of the 14,795 ANC register records reviewed, 100.0 % had the date of visit recorded, 99.1 % had age recorded, 87.8 % had the current HIV test result recorded, and 89.2 % had the patient’s HIV status recorded. Of the 13,926 records surveyed in ANC SS, 97.3 % had date of visit recorded, 99.4 % had age recorded, and 99.6 % had HIV test results from routine HIV testing recorded.

5. Assessment of WHO standard 5: State of routine HIV testing quality assurance practices

Eight (19.0 %) of 42 participating clinics failed to return PT results in the June 2010 PT round, and 13 (31.7 %) of 41 participating clinics failed to return results in the October 2010 PT round. Three (7.1 %) clinics the June round and 6 (14.6 %) clinics in the October round failed PT due to submission of incorrect test results. Two (4.8 %) clinics in the June round and three (7.3 %) clinics in the October round failed PT due to using an incorrect testing algorithm. One-third of clinics (33.3 %) in the June round and one-fifth of clinics (22.0 %) in the October round failed PT due to submission of incomplete testing information. Twenty-six (61.9 %) clinics failed PT for any reason in the June round, and 28 (68.3 %) failed in the October round.

## Discussion

We found that among 43 ANCs participating in ANC SS in Kenya, two of the five recommended WHO general standards for transitioning to a HIV antenatal surveillance system based on routine HIV screening were fulfilled. These suggested standards were (1) provision of routine antenatal HIV testing by all participating sentinel sites and (2) the presence of routine quality assurance for HIV testing. Conversely, the non-consent bias was too high and the quality of routinely collected antenatal data was too low to meet the suggested WHO criteria. Further, the agreement between individual-level test results from routine testing and UAT for the same patients fell below the WHO-recommended benchmark for high level of agreement between the two tests (4).

The comparability of HIV prevalence estimates based on data collected routinely in ANC registers and from ANC SS was similar to a smaller study conducted in six ANC SS sites in 2003 in which Seguy and colleagues reported that HIV prevalence obtained through ANC SS was 12.8 % compared to 14.4 % from ANC registers [5]. In a later study, Bolu and colleagues compared data from 43 ANC SS sites and corresponding ANC registers [6]. HIV prevalence obtained from ANC SS was 7.3 %. Among women who accepted routine antenatal HIV testing the prevalence was 8.0 %. HIV prevalence among those who declined or were not offered testing was 5.4 %.

Of great concern is that 24 % of women who tested negative during routine testing were positive on testing in ANC SS, resulting in a low positive percent agreement of HIV test results. Some of these discrepancies may have been the result of higher sensitivity of the fourth generation antigen-antibody EIAs used for ANC SS. Heavy patient load may have also led counselors to read HIV test results prematurely, resulting in women who tested HIV-negative in routine antenatal HIV testing who were in fact HIV-positive. As a direct result of this unexpected finding, the Kenyan Ministry of Health directed that, beginning in July 2010, all pregnant women testing HIV-negative in the first and second trimester be retested in the third trimester not only to detect intercurrent infection but also to identify HIV-positive women who were potentially misdiagnosed.

## Conclusions

In sum, our results suggest that while some of the WHO general standards to assess the ability of routine antenatal testing to be used for HIV surveillance have been met, others have not suggesting that Kenya is not yet in a position to transition to a surveillance system purely based on programmatic data. Encouragingly we found that HIV prevalence estimates obtained from routine antenatal HIV testing approximated HIV prevalence from ANC SS. However the low positive percent agreement between the routine and UAT HIV test results and poor PT performance suggest that routine evaluations of HIV testing data quality at ANC testing sites and review of central laboratory testing data for HIV surveillance are required before ANC SS can be discontinued. Since 2010, substantial improvements have been made in the quality of HIV programmatic data in Kenya which warrant a repeat assessment of the utility of routine antenatal HIV testing data for HIV surveillance. These results have broad implications in sub-Saharan Africa, where universal HIV testing of pregnant women is within reach. A standardized approach for evaluating the use of routine antenatal HIV testing for surveillance will ensure that countries can transition from conducting ANC SS when high quality programmatic data are available for decision-making.

## Abbreviations

ANC: Antenatal clinic
CI: Confidence intervals
IQR: Inter-quartile range
PDA: Personal data assistant
PMTCT: Prevention of mother-to-child transmission (of HIV)
PT: Proficiency testing
QA: Quality assurance
UAT: Unlinked anonymous testing
WHO: World Health Organization
